# Modeling and Electrochemical Characterization of Electrodes Based on Epoxy Composite with Functionalized Nanocarbon Fillers at High Concentration

**DOI:** 10.3390/nano10050850

**Published:** 2020-04-28

**Authors:** Antonino Cataldo, Giorgio Biagetti, Davide Mencarelli, Federico Micciulla, Paolo Crippa, Claudio Turchetti, Luca Pierantoni, Stefano Bellucci

**Affiliations:** 1 Department of Information Engineering, Polytechnic University of Marche, Via Brecce Bianche, 1, 60131 Ancona, Italy; 2 INFN-Laboratori Nazionali di Frascati, via E. Fermi 40, 00044 Frascati, Italy; 3 Qi technologies, Via Monte D’Oro, 2/a, 00040 Pomezia, Italy

**Keywords:** nanocomposite, electrical equivalent circuit, electrochemical impedance spectroscopy, graphene, carbon nanotubes

## Abstract

This paper deals with the electrochemical characterization and the equivalent circuit modeling of screen-printed electrodes, modified by an epoxy composite and loaded with carbon nanotubes (CNTs), pristine and functionalized NH_2_, and graphene nanoplates (GNPs). The fabrication method is optimized in order to obtain a good dispersion even at high concentration, up to 10%, to increase the range of investigation. Due to the rising presence of filler on the surface, the cyclic voltammetric analysis shows an increasing of (i) electrochemical response and (ii) filler concentration as observed by the scanning electron microscopy (SEM). Epoxy/CNTs-NH_2_ and epoxy/GNPs, at 10% of concentration, show the best electrochemical behavior. Furthermore, epoxy/CNTs-NH_2_ show a lower percolation threshold than epoxy/CNT, probably due to the direct bond created by amino groups. Furthermore, the electrochemical impedance spectroscopy (EIS) is used to obtain an electrical equivalent circuit (EEC). The EEC model is a remarkable evolution of previous circuits present in the literature, by inserting an accurate description of the capacitive/inductive/resistive characteristics, thus leading to an enhanced knowledge of phenomena that occur during electrochemical processes.

## 1. Introduction

Properties of carbon nanostructure are useful to obtain nanocomposites, characterized by excellent mechanical, electrical, and thermal properties far above those of base polymers, [1,2,3] and aimed at producing automotive components, medical devices, conducting plastics, lubricating additive, electromagnetic interference shielding/absorbers [4,5]. In order to give these outstanding properties, an accurate and engineered preparation method has to be selected. As a matter of fact, an appropriated process can allow obtaining a well disperse composite, avoiding the presence of an agglomerate of fillers that impact negatively the material properties [6]. Although the optimization of the preparation method may be performed, the nature of the interface between the filler and the matrix still plays an important role, so that the choice of a proper matrix, or the use of a dispersive agent, such as the surfactant, may be needed.

In order to improve the filler matrix interface, a no-covalent functionalization strategy can be used [7]. In particular, the use of a dispersive agent, e.g., melamine, is useful to avoid filler agglomeration and to create pure chemical bonds between matrix and filler, increasingly enhancing the electrical/mechanical properties. Among the plethora of polymeric matrices useful for composite fabrication, epoxy resin is one of the most studied matrixes. Epoxy resin is a well-known engineered thermosetting polymer with applications in electronics, anticorrosive coatings, marine, aerospace, and automotive systems. It has a better behavior than polyester, phenolic, and melamine resins, because it manifests no volatile loss and little shrinkage during the curing process, good chemical resistance and inertness, as well as versatility in selecting curing agents and conditions [8]. To make a more specific application example, epoxy resin nanocomposites are one of the most used nanocomposites in aeronautics and aerospace fields, due to the possibility to improve EMI shielding, and mechanical/electrical properties, while maintaining lightness [9,10,11].

For completeness, it is worth mentioning an additional remarkable property of nanocomposite materials, namely their electrochemical behavior, which allows the development of electrochemical sensors and energy storage devices, e.g., biological screening/detection and application to modified electrodes to enhance efficiency. The different carbon–carbon covalent bonds (hybridization) and organization of the carbon atoms into the structure of the nanocarbon allotropes determine different electrochemical properties. Consistently, from an electrochemical reaction kinetics point of view, it is known that the physical, chemical, and electronic properties of carbon electrodes have a dramatic effect on their electrochemical response.

In this contribution, we focus on carbon nanotubes (CNTs) and graphene (GNP) nanocomposites, that have been investigated in different electrode configurations by electroanalysis. As it holds for graphene [12], their unique and outstanding features, such as electrical conductivity, high aspect ratio, chemical stability, and mechanical strength, are enabling properties for many applications. For example, CNT-based sensors often enable higher sensitivity, lower limits of detection, and faster electron-transfer kinetics for target analytes as compared to traditional sensors based on bulk graphitic carbon electrodes. Nevertheless, a complex and detailed description of an equivalent electrical circuit is missing: To our best knowledge, only Randles circuit [13] or a parallel resistance–capacitance (RC) combination, and a variable number of RC series in parallel [14], were used so far to model the electrochemical behavior of nanocomposites.

Usually, carbon nanostructures are mixed with a binder (pasting liquid), so called carbon paste [15], with an alternative to carbon paste found in nanocomposites based on epoxy resin. 

The main aim of this paper is to propose a preparation method involving an ARV-310 vacuum planetary mixer (Thinky Corporation, Tokyo, Japan) in order to produce high quality and highly reproducible nanocomposites, reducing air bubbles, and increasing the homogeneity of the filler dispersion, even at a very high filler concentration. As known from the literature [16,17], the planetary centrifugal mixing action can help obtain a well dispersion in a short time and, operating at low pressure, to reduce the presence of air inside the matrix. 

More specifically, CNT and GNP as fillers and epoxy resins as the matrix are used according to a good interaction between the filler/matrix interface suggested by the Hansen Solubility Parameters (Epon 828 δ = 20 MPa^1/2^ [18], Graphene δ = 21 MPa^1/2^ [19], CNT δ = 19.5 MPa^1/2^ [20]). Furthermore, CNTs-NH_2_ are used to improve the filler/matrix interaction creating a chemical bond between each other. Finally, the above nanocomposites have been characterized both from microscopic and electrochemical points of view and have been successfully applied to obtain a modified screen-printed electrode. In addition, and in parallel to the above activity, we introduced a novel methodology for deriving a lumped electrical circuit element, which is electro-physically equivalent to the chemical process used in the synthesis of nanocomposite materials. With respect to a previous work [14], we developed a more accurate technique, based on MATLAB software, for the optimal extraction of the set of electrical parameters that best fit the measured impedance (resulting from the cyclic voltammetry analysis) in terms of modulus and phase, as functions of the frequency. In particular, given an assumed topology for the equivalent circuit derived from a chemical/physical understanding of the system, as described later, and that can possibly be composed of a relatively large number of components, each with multiple parameters to be estimated, a parametric model of the circuit was derived. The genetic algorithm implemented in the MATLAB optimization toolbox was then used to explore the large parameter space to find the optimal or near-optimal solution, which can then be further refined by a second-stage simulated annealing optimization.

Specifically, we i) added a contact resistance to simulate the liquid/composite interface, ii) replaced the RC series with a RCL series, for a better simulation of the current flowing in the fillers, and iii) included the hopping conduction mechanism (L) for CNT/epoxy, CNT-NH_2_/epoxy, and GNP/epoxy nanocomposites [21,22,23].

## 2. Materials and Methods 

For the preparation of nanocomposite materials, the commercial epoxy resin EPON™ 828, a medium-low density resin marketed by Hexion, was used as a matrix. Tetraethylene pentamine, modified with the addition of formaldehyde thanks to a nucleophilic addition reaction, was used as a curing agent, called A1 [24,25].

Commercial CNTs (Heji) and CNT-NH_2_ (NANOLAB) were used as fillers. The former nanotubes have an external diameter between 8 and 15 nm, the latter have an external diameter of 15 ± 5 nm, and both have a degree of purity greater than 95%.

The graphene nano platelets (GNPs), instead, were prepared in the laboratory starting from the Asbury^®^ expandable graphite, of 99.1% purity and containing 3.508% sulfur, by means of the microwave-assisted thermal expansion technique [11,26].

The graphite is placed in a special ceramic crucible and inserted into a homemade microwave oven, which operates at a power of 800 W. This technique requires that the substances intercalating the graphite decompose quickly, causing their expansion thanks to the thermal shock suffered by the material. During the microwave treatment, the intercalated substances start to decompose at a temperature of about 140 °C (±20 °C). The developing gases modify the dielectric constant of the air to the point that the electric field of the microwaves exceeds the dielectric strength generating a voltaic arc. This causes a sudden temperature increase of over 1000 °C, further decomposing the intercalating substances and self-feeding the process. This rapid thermal shock is necessary to ensure good material expansion. The expansion process ends in about 10 s [27].

After weighing the appropriate amounts of the two components, the dispersion of the carbonaceous filler in the matrix started with an ultrasonic tip. The mixing of the two phases is continued, to ensure a good homogeneity of the sample, with two programs of the Planetary Vacuum Mixer (ARV-310 Thinky Corporation). These steps were also necessary to eliminate the air bubbles present in the epoxy resin, which would compromise the homogeneity of the nanostructured composite [10]. Afterwards, the curing agent (22% by weight of the composite) A1 is added and the third and final program of the ARV-310 is started, which is used to mix the hardener with the composite and to complete the elimination of the air bubbles. Finally, the obtained composite is left to rest in the air for more than 20 h. This procedure was used to produce the nanocomposite at 0.25%, 1%, and 2%.

Since at a 10% loading level of nanofillers the composite immediately becomes extremely viscous and sonication with the tip becomes practically impossible. Furthermore, as the viscosity increases, mixing with a planetary mixer improves the efficacy. Therefore, we replace the ultrasounds tip with a supplementary step in ARV-310 to obtain a good homogeneity of mixture, as demonstrated in a previous work [17]. In fact, the ARV-310 generates a big centrifugal force during the planetary motion that is able to mix and de-aerate at the same time. In addition to this, ARV-310 has a rotary pump to help the removal of the air contained in the mixture. Inside the sample holder, shear stresses are generated, thus permitting to mix and homogenize two different phases. These shear stresses are directly linked with the viscosity of the medium: The higher is the viscosity, the higher are the shear stresses and more efficient the mixing is, and so is the air evacuation. In the case of a high amount of filler, 10%, the ARV-310 works as the most efficient method of mixing.

We chose this range of concentrations because:

- The 0.25%, 1%, and 2% concentrations were investigated earlier with different techniques [9], they are close to the percolation threshold;
- In the nanocomposite fields, it is quite hard to obtain good dispersed samples at a high concentration. By means of ARV-310, we could overcome this limitation and probe the high filler content composite properties.

Even so, it is too difficult to go above 10% due to excessive viscosity of the matrix, which does not allow for the realization of homogeneous dispersion of the filler.

Finally, we modified a screen-printed electrode (SPE) DRP-110 (available from Dropsens) with a drop of each nanocomposite to obtain a new SPE in which it should be possible to measure the electrochemical properties of each nanocomposite.

In order to study the electrochemical behavior of the modified SPE versus the loading of filler, cyclic voltammetry (CV) characterization was performed. K_3_[Fe(CN)_6_] was used as detectable electrochemical species, at a concentration of 5 mM in KCl 0.01 M. The CV were carried out in the range of −0.1–0.6 V, with a scan rate of 100 mV/s.

### 2.1. Theory and Calculation

#### Equivalent Electrical Circuits

**Figure 1:**
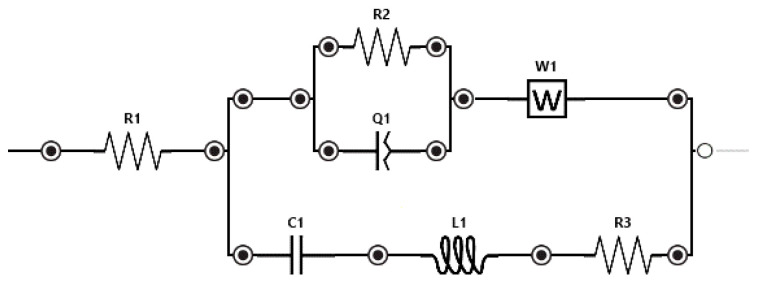
Equivalent electrical circuit used for fitting.

From the experimental data obtained by electrochemical impedance spectroscopy, the parameters of an electrical model can be determined. To this end, an equivalent electrical circuit can be used, which is a refinement of that proposed in [14], as previously stated. In this refinement, we introduce the “constant phase elements”, which have a frequency-dependent admittance given by Y(ω) = ω_0_ C(j ω⁄ω_0_)^N^ where C and N are parameters. C is the equivalent capacitance at an arbitrary frequency ω = ω_0_, and N, with 0 < N < 1, determines the phase shift. The modulus of the admittance increases with frequency at a fixed rate of 20 N dB per decade, while the displacement is -N π⁄2 at all frequencies, hence the element name. The particular cases N = 0 and N = 1 correspond to a standard resistor and capacitor, respectively, while the case N = 1⁄2 is known as the *Warburg element*, useful to model the diffusion of a liquid inside a matrix. In Figure 1, we denote the “constant phase element” by Q1 = ω_0_ C2(j ω⁄ω_0_)^N2^, whereas the remaining “constant phase element” W1 is obtained from Q2 = ω_0_ C3(j ω⁄ω_0_)^N3^ in the particular case N3 = 1/2.

To perform the fitting of the parameters, it is necessary to define a distance function that describes how much the circuit response is (dis)similar to the measured data. The standard Euclidean distance was deemed inappropriate given the fact that measured data span multiple orders of magnitude. Hence, we used the distance:

(1) $$d=\underset{\omega }{\sum }log_{10}\left(\left|x\left(\omega \right)\right|\right)+w\left(\omega \right)\left|I\left(log_{10}\left(x\left(\omega \right)\right)\right)\right|$$

where

(2) $$x\left(\omega \right)\;=\;\frac{Z\left(\omega \right)}{Z_{e}\left(\omega \right)}$$

is the ratio of measured impedance *Z(ω)* to modeled impedance *Z_e_(ω)*, and *w(ω)* is a weighting factor to balance amplitude matching versus phase matching *w(ω)* was empirically set to a starting value of 10 at low frequencies and then decreased to 1 for frequencies greater than 2 kHz, to account for less accurate phase measurements at these higher frequencies.

## 3. Results

As previously demonstrated [17], the realization process, carried out by the ARV-310 planetary mixer, is powerful to prepare nanocomposite materials of high quality dispersion: The scanning electron microscopy (SEM) characterization, carried out by means of a Tescan Vega II microscope, highlights a good dispersion of all types of filler in the investigated range.

**Figure 2:**
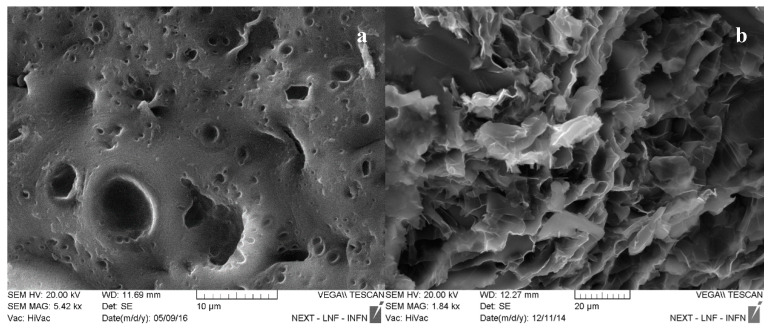
SEM image of the graphene nanoplate (GNP)/epoxy composite loaded at 2%w/w (**a**) and loaded at 10%w/w (**b**).

**Figure A5:**
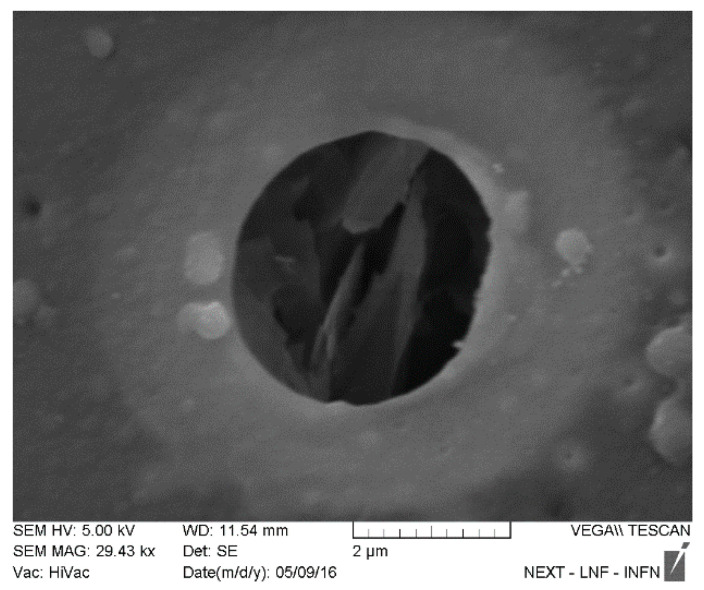
SEM image of GNP/Epoxy composite loaded at 2%w/w_._

In Figure 2, the surface of GNP composites at 2% and 10% were selected to highlight the differences of the surface:

At low concentration, no regions of agglomerate fillers are visible (Figure 2a). Furthermore, the surface of the nanocomposite appears with a high porosity in which nanofillers protrude from the holes (see Appendix A, Figure A5). The nanocomposite loaded at 10% of filler shows the analogous features, such as no agglomerates and porosity, besides an outstanding presence of filler on the surface, according to the amount of loading. The increase of superficial area, due to the presence of filler, is most evident in these nanocomposites.

These surface properties, porosity, and nanofiller emerging from the pores, could influence the electrochemical behavior of the nanocomposite as an electrode and increase the superficial area of SPE modified with nanocomposites with respect to the SPE modified with epoxy resin. In fact, the superficial area plays an important role in the electrochemical process: The greater the superficial area is, the greater the number of redox processes that occur on the electrode surface.

The nanocomposites were studied from an electrochemical point of view using cyclic voltammetry (CV).

**Figure 3:**
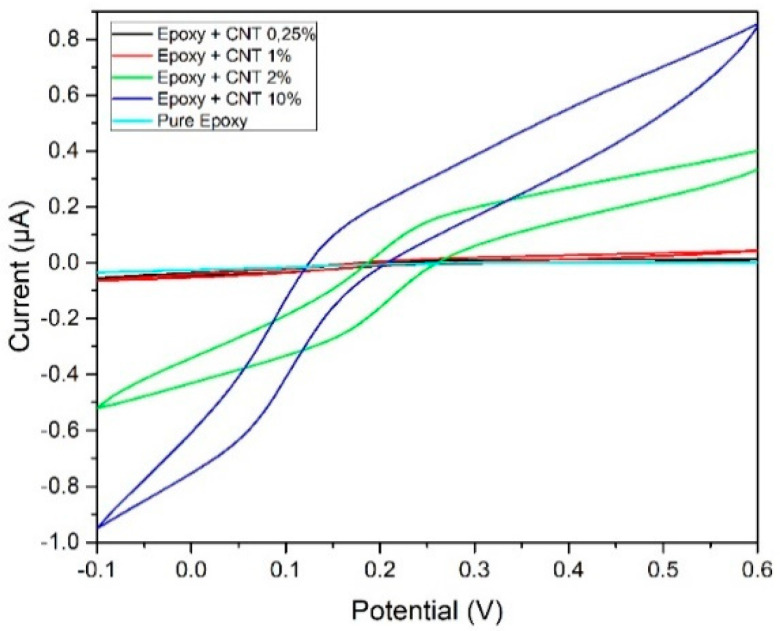
Cyclic voltammograms obtained by the screen-printed electrode (SPE) modified with epoxy and carbon nanotubes (CNT) nanocomposites at different filler concentrations.

Firstly, we analyze the cyclic voltammetric behavior of nanocomposites having pristine carbon nanotubes as a filler. The cyclic voltammograms are reported in Figure 3 for each filler concentration.

From these cyclic voltammograms, the CV trend is visible but there are no peaks related to the reduction and oxidation of the analyte (Fe^2+^/Fe^3+^). The comparison among the pure resin and nanocomposite filled with CNT showed an increase in the conductivity of the nanocomposites as the concentration of the nanofiller increases. Furthermore, it is possible to distinguish the percolation threshold between 1% and 2%, in good agreement with previous results using other techniques (1.5% reported in [9]). 

To verify the stability and improve the response of the samples, we proceeded by placing the SPE in water in order to condition them and acquire the voltammetric measurements after various time intervals (up to three days).

The conditioning improves the response of the composites to the electrochemical stimulus by presenting the peaks to the discharge potential of the electroactive species used. In particular, it is interesting to note that the conditioning time decreases as the concentration of the nanofiller present in the matrix increases, observing the shorter time in the nanocomposite, loaded at 10%.

**Figure 4:**
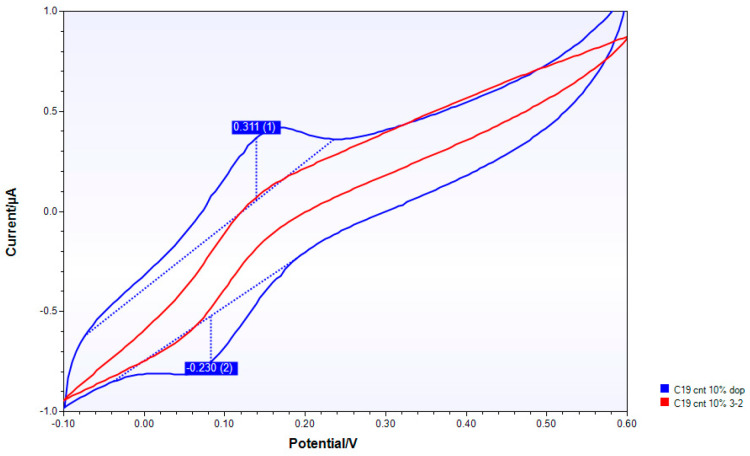
Cyclic voltammetry (CV) of CNT/epoxy composite, 10%, before (red curve) and after 3 h (blue curve) in water.

In Figure 4, the CVs show the behavior of the nanostructured composite, loaded at 10% of CNT pristine, before and after conditioning. The conditioning time in water is really shorter, 3 h, than the conditioning time for the other nanocomposite, three days. Thus, it could suggest an important role of water percolation in the electrochemical processes: In fact, this could be the effect of the superficial swelling and absorption of water that allows a more efficient electrochemical ion exchange.

**Figure 5:**
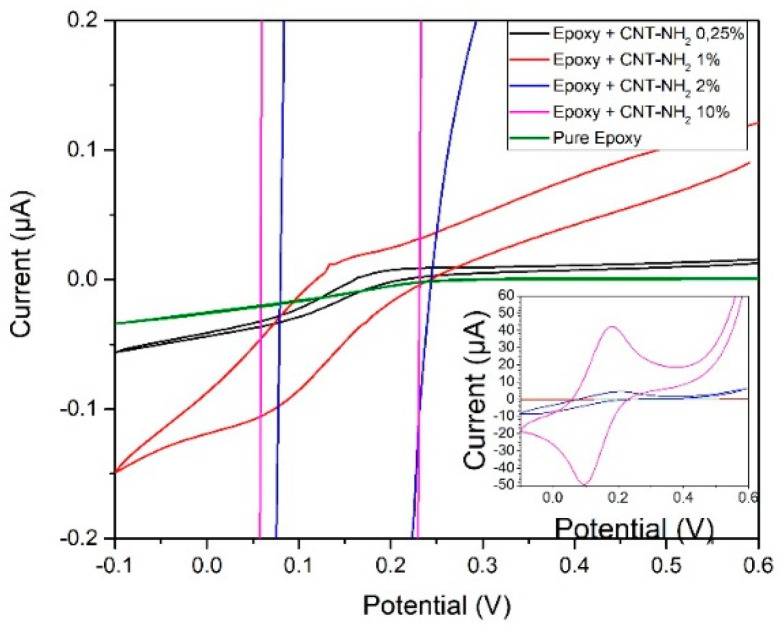
Cyclic voltammograms obtained by the SPE modified with epoxy and CNT-NH_2_ nanocomposites at different filler concentrations.

Then, we analyze the behavior of nanocomposites having carbon nanotubes functionalized with NH_2_ groups as fillers. The cyclic voltammograms are reported in Figure 5 for each concentration of the filler.

**Scheme 1:**
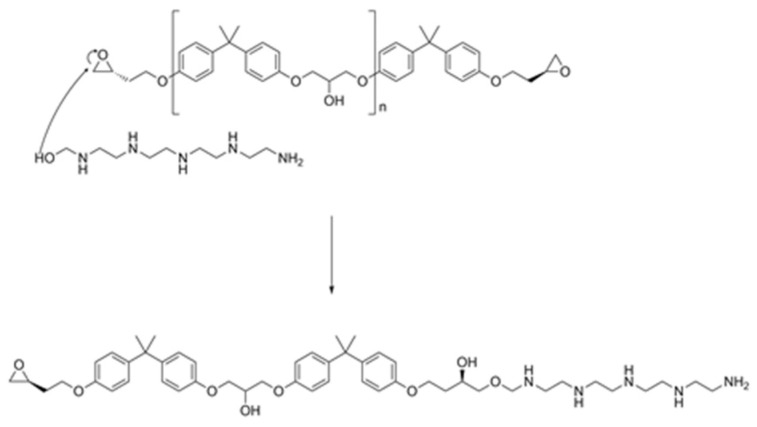
Curing process in which a nucleophilic group (hydroxyl or amine) is involved in the epoxy ring opening.

As visible from the curves reported in Figure 5, the composites having the CNT-NH_2_ nanofiller show the classic trend of a CV and present peaks related to the redox process, starting from 2% and well visible in the 10% nanocomposite. Furthermore, it is possible to distinguish the percolation threshold also in these nanocomposites: In this case, the percolation threshold is considerably lower than the percolation threshold of CNT pristine nanocomposite. In fact, the percolation threshold is estimated below 1%. This phenomenon could be explained by analyzing the curing process of the epoxy resin, as shown in Scheme 1.

In this scheme, the opening mechanism of the epoxy ring by means of a curing agent (A1 [24]) is reported, in particular by means of a nucleophilic group, in this case the hydroxyl or amine group present in the A1. 

**Scheme 2:**
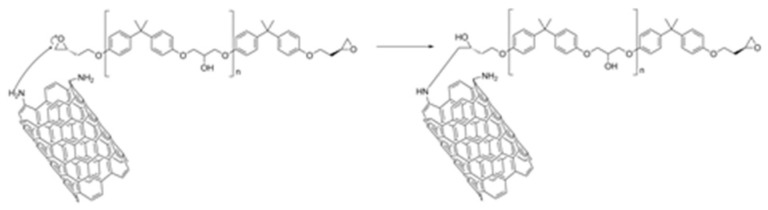
Curing process in which an amine group on the edge of CNT-NH_2_ is involved in the epoxy ring opening.

The reactivity of the epoxy ring is obviously connected by nucleophilicity of functional groups and also by steric hindrance. Therefore, the amine group present on the surface of a carbon nanotube could react and open the epoxy ring if not sterically hindered. Probably, the amine group present on the edge of CNT-NH_2_ could be in the best position to open the epoxy ring [28], as shown in Scheme 2.

In this hypothesis, a chain of epoxy resin is directly linked to the carbon nanostructure, as represented in Scheme 2, and could more efficiently enhance the conductivity pathway.

Analogously as CNT/epoxy nanocomposites, we proceeded in placing the SPE in water in order to condition them and acquire the voltammetric measurements after various time intervals (up to three days).

**Figure 6:**
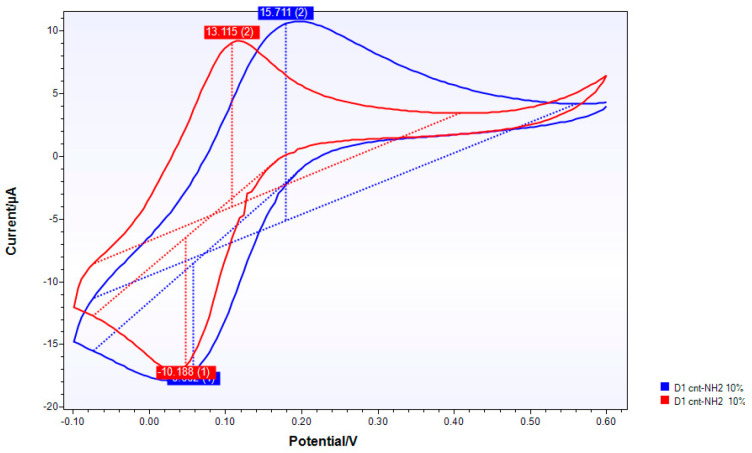
CV of the CNT-NH_2_/epoxy composite, 10%, before (red curve) and after 1 h (blue curve) in water.

**Figure A13:**
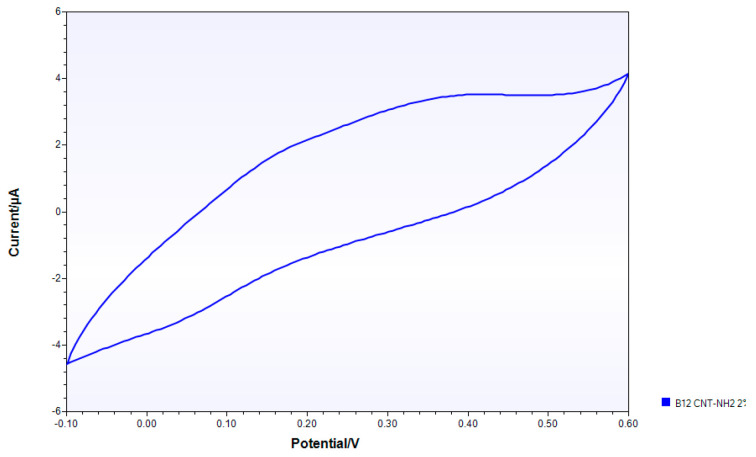
CV of CNT-NH_2_/epoxy composite, 2%, after 3 days in water.

Moreover, although in this case the conditioning process improves the electrochemical response of SPE, i.e. Figure 6, the process seems not to be dependent on the concentration. In particular, composites loaded at 0.25% and 1%_w/w_ of CNT-NH_2_, show a marked improvement in the response to electrochemical stress (see Appendix B). On the other hand, the composite obtained at a concentration of 2% shows an improvement in conductivity, but the peaks, related to the discharge potential of the electroactive species used, appear broad, not useful for the detection purpose (see Appendix B, Figure A13).

The cyclic voltammograms of the 10% composite of CNT-NH_2_ show the best electrochemical response in this series, as it responds very well to electrochemical stress showing the discharge signal of the electroactive species. The improvement of the electrochemical response of SPE strengthens the suggestion of a superficial water absorption on the electrode interface, the importance of water percolation, and confirms the superficial swelling and absorption water.

**Figure 7:**
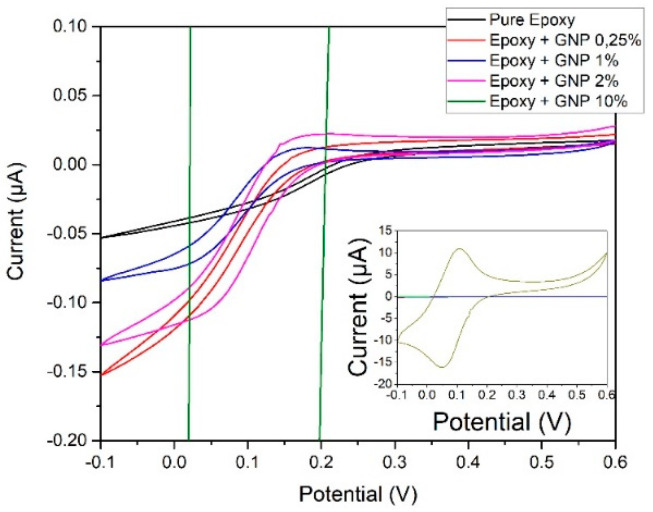
Cyclic voltammograms obtained by the SPE modified with epoxy and GNP nanocomposites at different filler concentrations.

Finally, we analyze the behavior of nanocomposites having graphene nanoplates as fillers. The cyclic voltammograms are reported in Figure 7 for each filler concentration:

Even for these nanostructured composites, at low concentrations of carbon nanofiller, no significant peak is obtained without conditioning. Nevertheless, the percolation threshold is determined between 2% and 10%.

We carried out the conditioning in water for these nanocomposites, too.

Even for composites with GNP as the filler, the conditioning step greatly improves the electrochemical sensitivity. In this case, it is interesting to highlight that the necessary conditioning time decreases as the concentration of the nanofiller dispersions in the polymer matrix increases.

**Figure 8:**
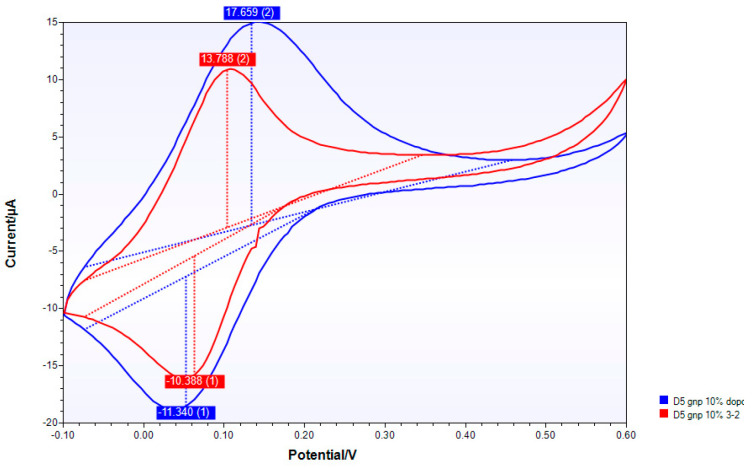
CV of the CNT-GNP/epoxy composite, 10%, before (red curve) and after 1 h (blue curve) in water.

From cyclic voltammograms, in Figure 8, we can see how the electrochemical response of the nanocomposite enhances after conditioning, even if the peaks relative to the redox process are just present before the conditioning step.

Then, we would argue that the conditioning step influences the electrochemical properties of the modified SPE: We suppose that the improvement of the electrochemical properties is due to the water percolation onto the electrode surface. The water, absorbed in the inner layers of the nanocomposite, promotes the electrochemical processes, previously restricted partially by the epoxy resin. The presence of carbon nanofillers mitigates the epoxy restriction and, in the case of highest concentration, promotes, in turn, the redox reactions due to the marked presence at the surface.

Since the conditioning step has an important role in the electrochemical behavior and the best electrochemical response is present in nanocomposites loaded at 10% of the filler, we studied the electrochemical impedance spectroscopy (EIS) to analyze and model the equivalent electrical circuit, justifying the electrochemical behavior and highlighting the circuit elements responsible for the working principles.

The screen-printed electrodes with 10% filler (MWCNT pristine, NH_2_ and GNP) were characterized by electrochemical impedance spectroscopy, before and after immersion in water.

**Figure 9:**
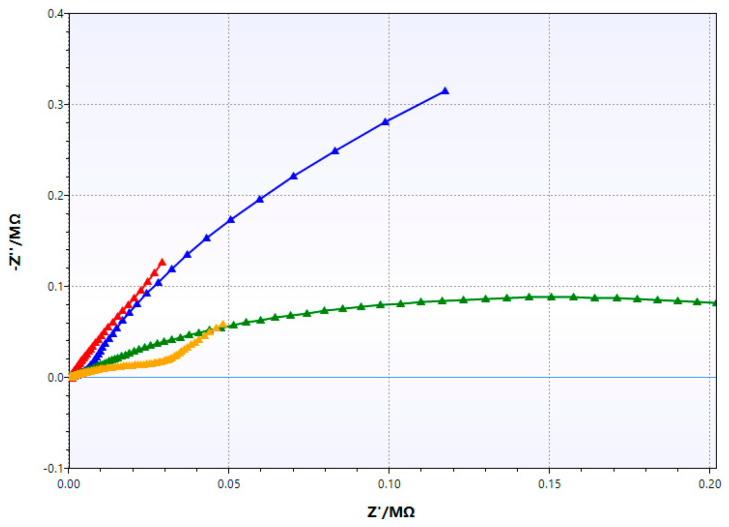
Nyquist plots of neat epoxy resin before (blue curve) and after (red curve) conditioning and CNT/epoxy 10% before (green curve) and after (orange curve) conditioning.

**Figure 10:**
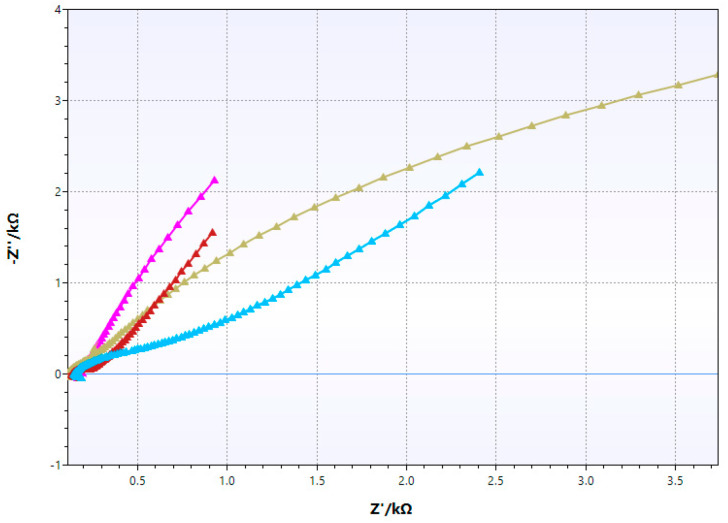
Nyquist plots of CNT-NH_2_/epoxy 10% before (pink curve) and after (grey curve) conditioning and GNP/epoxy 10% before (brown curve) and after (light blue curve) conditioning.

The Nyquist plots are shown in Figure 9 and Figure 10.

The Nyquist plots show a complex pattern of impedance, not attributable to a classical Randle’s circuit.

In order to provide a compact and efficient model of the overall process, we investigate and propose an equivalent lumped elements electrical circuit, that, on the one hand, is based on a previous article of the literature [14], and, on the other, provides a remarkable and definite improvement of it in terms of applicability. In fact, the general aim is also to use the equivalent circuit as a design tool for the optimal setup of the global chemical process. In the following, we will make use of the concepts introduced in Section 3.

In Reference [14], a parallel RC combination (modeling the epoxy resin) and a variable number of RC series in parallel to the previous one (modeling the fillers network) were considered to achieve a good fit between the network and the measured equivalent impedance. 

In this work, in order to refine the base equivalent electrical circuit, we decide to modify the circuit by adding the contact resistance to simulate the liquid/composite interface and replacing the RC series with an RCL series to simulate the mechanisms of current flow in the fillers, including the hopping conduction mechanism (L) [21,22,23].

Finally, since the CV characterization showed an important role of the conditioning step, we decide to add the Warburg element, which takes into account the diffusion of a liquid inside the matrix. In fact, it is possible to demonstrate that the Warburg impedance, defined by:

(3) $$Z_{W}=\frac{1}{\sigma \sqrt{i\omega }}$$

is correlated with the ion diffusion in the electrochemical system, considering the second Fick’s law and defining the “Warburg capacitance” as:

(4) $$\sigma =\frac{FA}{E}\underset{i}{\sum }z_{i}\sqrt{D_{i}}\left(c_{i}^{0}-c_{i}^{b}\right)$$

in which *F* is the Faraday constant, *E* is the potential difference between the working and counter electrodes, *A* is the unit area of electrode, *z_i_* is the ion charge, *D_i_* is the diffusion coefficient, and *c_i_* is the concentration of ions [29].

**Figure 11:**
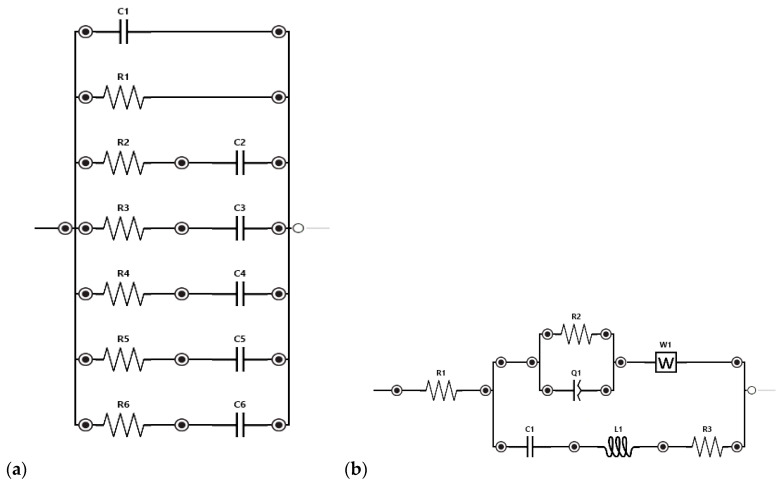
Comparison between the old [14], (reproduced with permission from Nanotechnology, IEEE Transactions on 24 April 2020) (**a**), and new (**b**) equivalent electrical circuit.

As it is possible to see in the comparison of Figure 11, the equivalent electrical circuit proposed in the present work is simpler and more precise than previously proposed. In Figure 11, we define Q1 = ω_0_ C2(j ω⁄ω_0_)^N2^.

The results of the fitting performed using an optimization toolbox for MATLAB are reported in Table 1; we use the formula Q2 = ω_0_ C3(j ω⁄ω_0_)^N3^ where N_3_ was fixed at 1⁄2, being Q2 = W1 a Warburg element. The best fitting parameters confirm the improvement of electrochemical properties given by loading of the nanofiller: In fact, it is possible to observe a general decreasing of values of resistance with respect to the neat epoxy resin and an increase in capacitance values.

**Figure 12:**
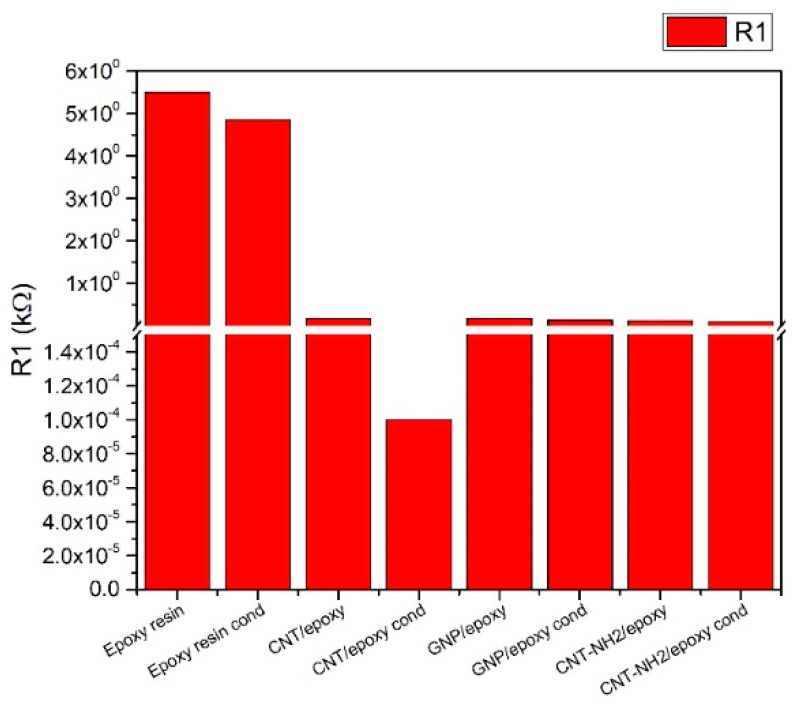
Best fitting values of resistance liquid/composite interface (R1) for all samples.

In particular, the resistance at the liquid/composite interface is sensibly decreased, as shown in Figure 12.

The decreasing in resistance allows the charge/discharge processes to the SPE. However, this parameter is not enough to guarantee that redox processes occur at the SPE. In fact, beyond the reduced resistance at the liquid/composite interface, it is essential that a conductive pathway occurs and promotes the electron migration from the SPE bulk to the redox species. The inductance (L1) and all capacitive elements (C1, C2, and W1) could suggest the electronic pathway.

**Figure 13:**
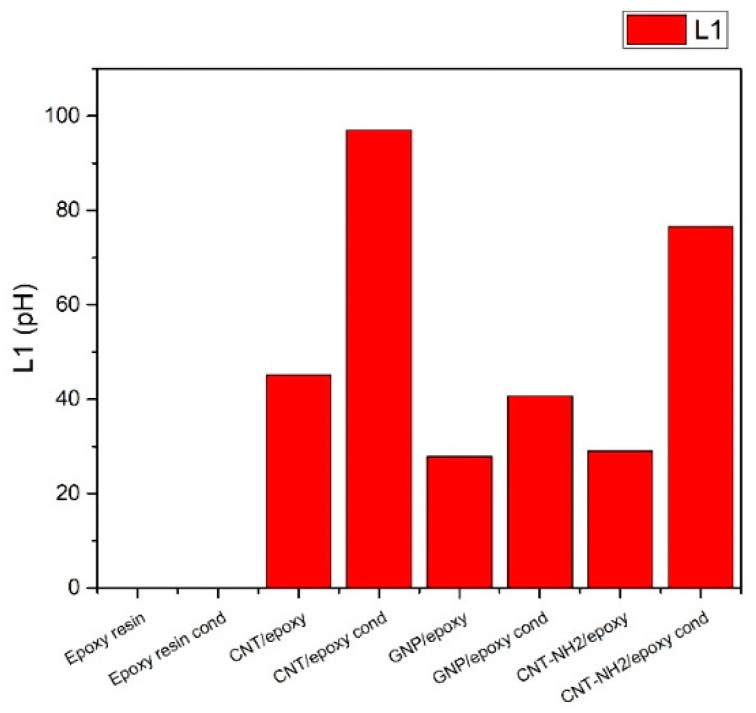
Best fitting values of electronic hopping (L1) for all nanocomposites.

In fact, the inductance models the electronic hopping between different nanofillers or the matrix and nanofiller [21,22,23]. In the nanocomposite, the inductance increases after the conditioning step, as shown in Figure 13.

**Figure 14:**
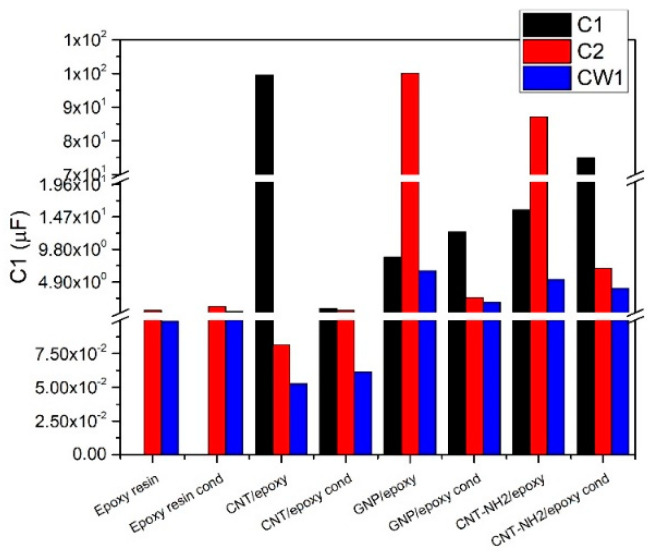
Best fitting values of filler capacitance (C1), matrix capacitance (C2), and Warburg capacitance (CW) for all samples.

After conditioning, we observe an increment of more than 50% for nanocomposites loaded with CNT and CNT-NH_2_, and an increment of 30% for nanocomposites loaded with GNP. This enhancement could be attributed to the water diffusion inside the pores of the matrix. Furthermore, we can notice that the best fitting values of the CNT/epoxy nanocomposite are higher than both one of the CNT-NH_2_/epoxy and GNP/epoxy, contrary to the results of CV characterization, in which the CNT-NH_2_/epoxy and GNP/epoxy work better than the CNT/epoxy one. For a complete explanation of this electrochemical phenomenon, we analyze the trend of capacitive elements, as shown in Figure 14.

The best fitting values obtained for the capacitive elements show the highest capacitive contribution for the CNT-NH_2_/epoxy and GNP/epoxy with respect to the neat epoxy resin. The sum of capacitance of the system (C1, related to filler capacitance; C2, related to matrix capacitance; Warburg capacitance, related to water and ion diffusion inside the matrix) can explain the good behavior as an electrode for CNT-NH_2_/epoxy and GNP/epoxy systems. Therefore, the conditioning step, as observed during CV characterization, has a real positive effect on the electrochemical behavior: In fact, the conditioning step affects all capacitances since the diffusion of water in the matrix changes the response of nanocomposites and, furthermore, allows the ions to penetrate inside the matrix and to have a higher electrochemical area for redox processes, as shown by the values of Warburg capacitance. On the other hand, the Warburg capacitance values for CNT-NH_2_/epoxy and GNP/epoxy systems are in good agreement with CV characterization: In fact, we observe a low improvement after the conditioning step for these systems. A possible explanation could be the high electroactive area due to the nature of the fillers: In fact, if GNPs have a great superficial area, thanks to their aspect ratio, that protrude dramatically at the SPE surface, the CNTs-NH_2_ play a role together with the matrix, since they are linked directly with the epoxy chain, i.e., Scheme 2, obtaining some conducting region of epoxy resin and hence increasing the electroactive area.

## 4. Discussion

The analysis provides various and interesting results: We can state that the response to the electrochemical stimulation of these nanocomposites improved with the increase in the concentration of the filler used. It is hypothesized that this improvement can be attributed to the increasing presence with the concentration of nanocharges on the surface of the electrode, as observed in the SEM images. These surface nanostructures greatly increase the electrode area, a crucial parameter for electrochemical measurements, expanding the electrode-solution interface. Moreover, the remarkable conductivity of these nanostructures promotes the redox processes and the current passage generated by them.

**Figure 15:**
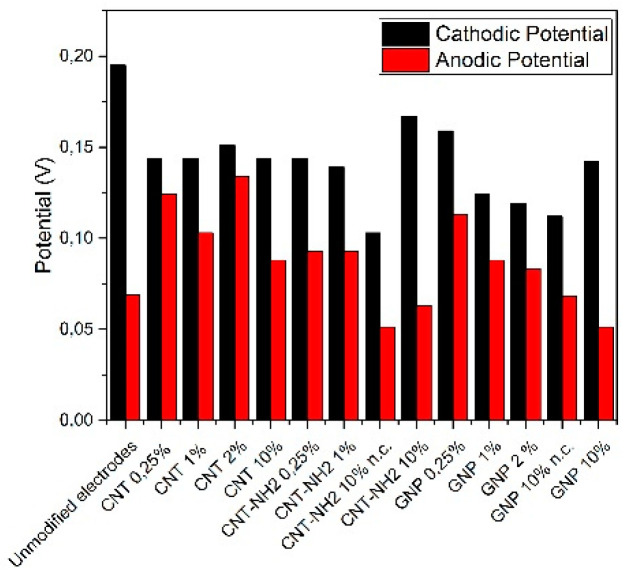
Potential redox values of modified SPE.

**Figure 16:**
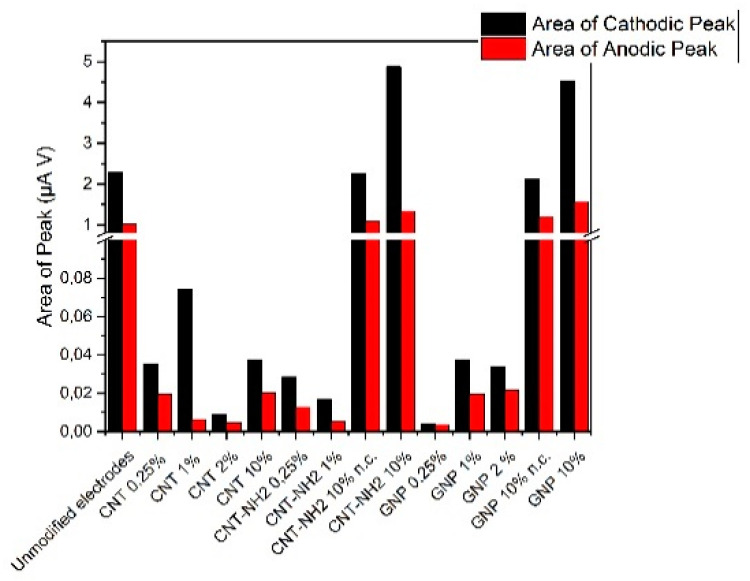
Peak area values of modified SPE.

**Figure 17:**
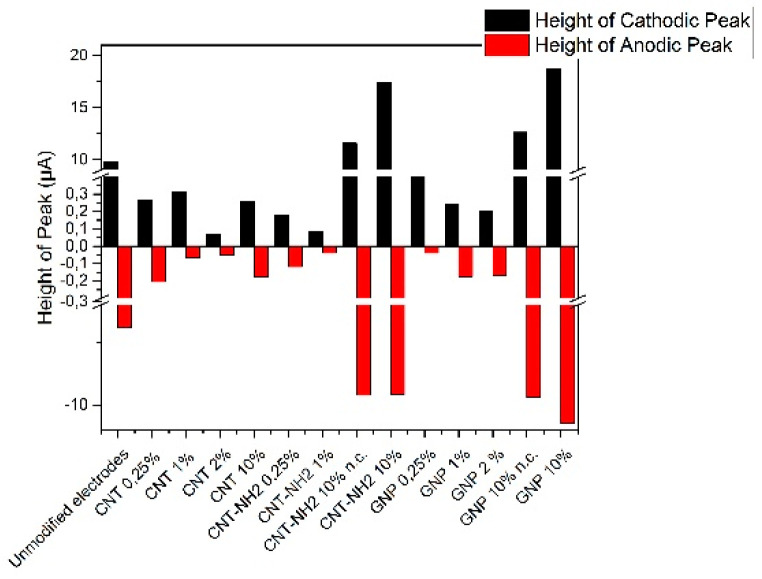
Height peak values of modified SPE.

**Figure 18:**
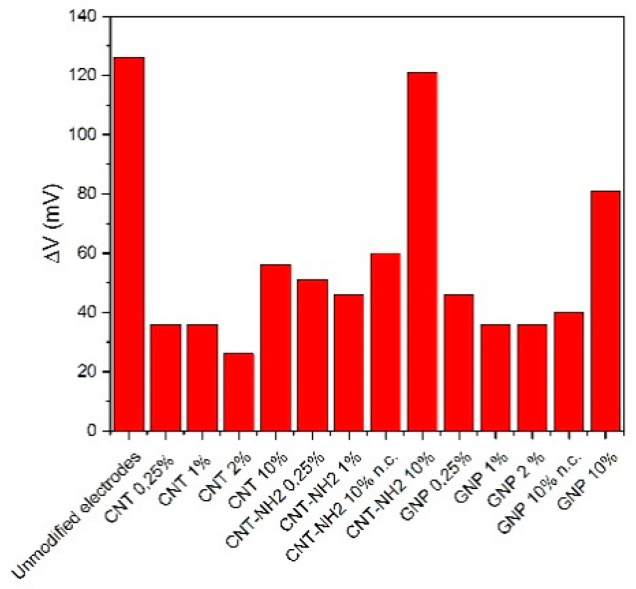
Potential redox (ΔV) values of modified SPE.

Another experimental evidence shows that, for the composites having medium-low concentrations, the conditioning step is necessary. This is because the epoxy resin used, being hydrophobic, requires a certain amount of time for the water to penetrate and reach the nanostructures (which for low concentrations we do not find an appreciable quantity on the surface). The conditioning, thus, facilitates the passage of the analyte (present in the aqueous solution) from the solution to the surface of the composite and the achievement of this by the nanocharges, allowing the oxidation/reduction process. The characteristics of the modified electrodes are summarized in the following graphs in which the values of potential, area, height of the peak, and the difference between the cathodic and anodic peaks of each composite are reported (Figure 15, Figure 16, Figure 17 and Figure 18).

The graphs show that the electrodes modified with 10% CNT-NH_2_ and GNP composites have better properties than the other modified electrodes and similar values to the unmodified electrode.

From the cyclic voltammetric analysis, it is possible to deduce that the composites having the functionalized nanotube-filler (-NH_2_) respond to the electrochemical stimulus better than the others. A possible justification for this behavior concerns precisely the functionalization present on the nanotubes. In fact, it makes the nanostructures more polar, thus increasing the wettability of the composite; moreover, thanks to the amine functionalization, in the crosslinking step of the composite, the epoxy resin rings, once opened, bind directly to the nanostructure, lowering the percolation threshold of the composite.

**Figure 19:**
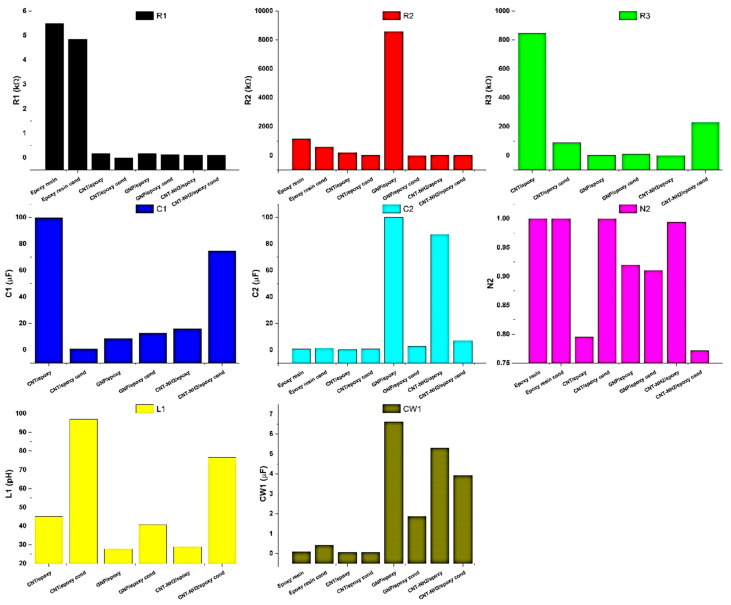
Best fitting parameters obtained by nonlinear regression using the proposed equivalent electrical circuit.

The CV analysis is supported by the EIS characterization and relative modeling of an equivalent electrical circuit. A dedicated equivalent electrical circuit is proposed in which resistance to the liquid/composite interface and water diffusion phenomenon (Warburg capacitance) are taken into account. All best fitting values are summarized in Figure 19.

The best fitting parameters of an equivalent electrical circuit, performed using the optimization toolbox for MATLAB, confirms the observation during the CV analysis and SEM characterization, revealing the importance of the water diffusion phenomenon and increased surface electrode area. This refined and elegant equivalent electrical circuit is able to demonstrate and confirm the previous analysis, simplifying the old circuit present in the literature and enriching the knowledge of phenomena that occur during electrochemical processes.

## 5. Conclusions

This paper proposes a preparation method involving a vacuum planetary mixer (ARV-310, Thinky Corporation) to produce a high-quality nanocomposite, reducing air bubbles, at a very high filler concentration, up to 10%. The microscopic characterization confirms a good filler dispersion inside the matrix and an increase in surface area due to the presence of filler at the air/composite interface, especially at high concentration. The screen-printed modified electrodes, obtained using the nanocomposites, show a good electrochemical behavior due to the increase in surface area and the electrical properties of the fillers. Moreover, we demonstrate from the electrochemical point of view that nanocomposites based on a 2D filler work better than the ones based on a 2D filler due to the better filler/matrix interaction. In addition, we demonstrate that a direct chemical bond (NH_2_) between the filler and matrix is more effective than the filler/matrix interaction. Finally, the new proposed equivalent circuit model analyzes in detail all the possible electrical processes that can occur. This result allows engineering of the nanocomposite to enhance a particular electrical property in spite of others.

For some future research directions, a change of the polymer matrix could be useful to test the model and to engineer specific new nanocomposites. Furthermore, the use of GNP-NH_2_ should be investigated to confirm the improvement of nanocomposite properties due to a direct chemical bond. The use of nanocomposites is one of the key developments of technological platforms for a broad range of applications and operating frequencies, covering the radio-frequency (RF) spectrum, up to the microwave/mm-wave region [30].

## Figures and Tables

**Table 1:** Best fitting parameters obtained by nonlinear regression using the proposed equivalent electrical circuit in Figure 1.

	R1	R2	R3	C1	C2	N2	L1	W1
	[kΩ]	[kΩ]	[kΩ]	[µF]	[µF]		[pH]	[µF]
Epoxy resin	5.50	1137.1	-	-	0.57	1.00	-	0.10
Epoxy resin cond	4.84	595.0	-	-	1.18	1.00	-	0.42
CNT/epoxy	0.17	197.7	844.69	99.50	0.08	0.79	45.11	0.05
CNT/epoxy cond	0.00	7.9	89.14	0.93	0.56	1.00	96.96	0.06
GNP/epoxy	0.17	8584.0	1.37	8.61	99.99	0.92	27.82	6.60
GNP/epoxy cond	0.13	0.18	8.62	12.42	2.52	0.91	40.69	1.86
CNT-NH_2_/epoxy	0.12	9.14	0.24	15.78	87.16	0.99	29.00	5.29
CNT-NH_2_/epoxy cond	0.10	6.57	229.42	74.87	6.94	0.77	76.54	3.94

**Figure A1:**
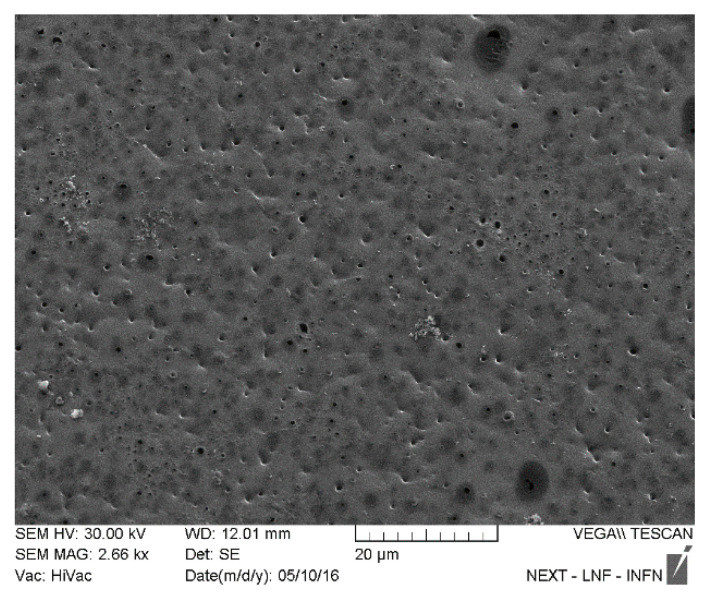
SEM image of CNT/Epoxy composite loaded at 2%w/w_._

**Figure A2:**
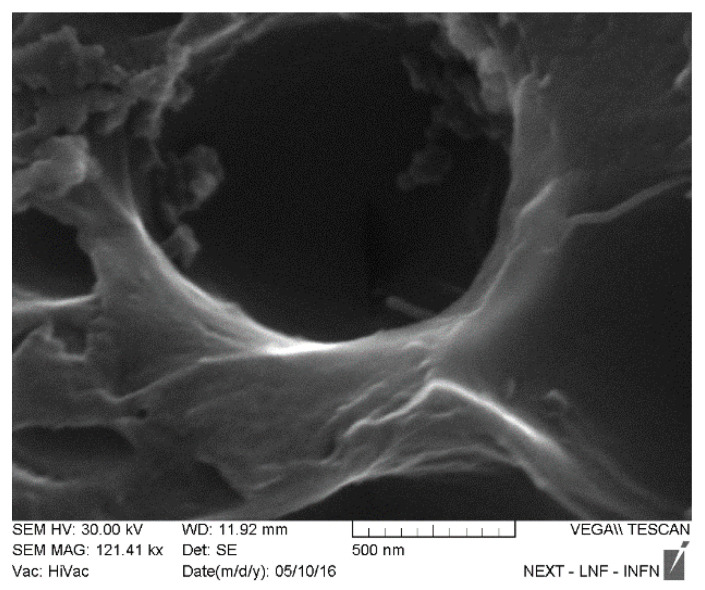
SEM image of CNT/Epoxy composite loaded at 2%w/w_._

**Figure A3:**
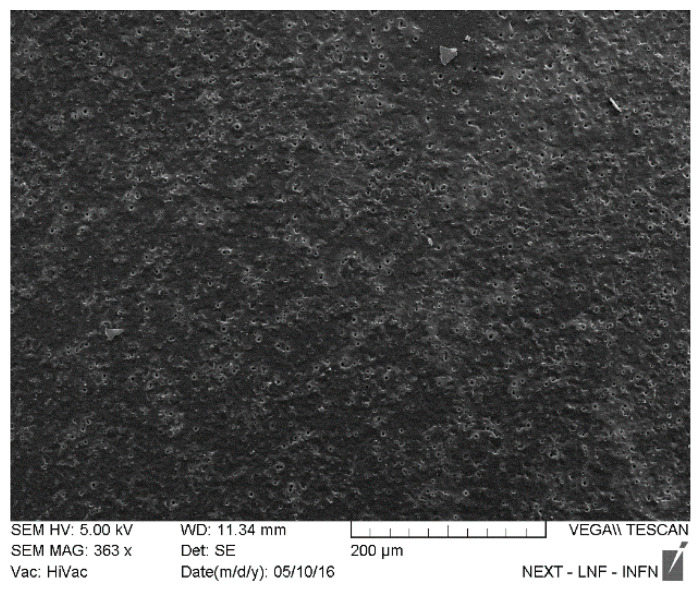
SEM image of CNT-NH2/Epoxy composite loaded at 2%w/w.

**Figure A4:**
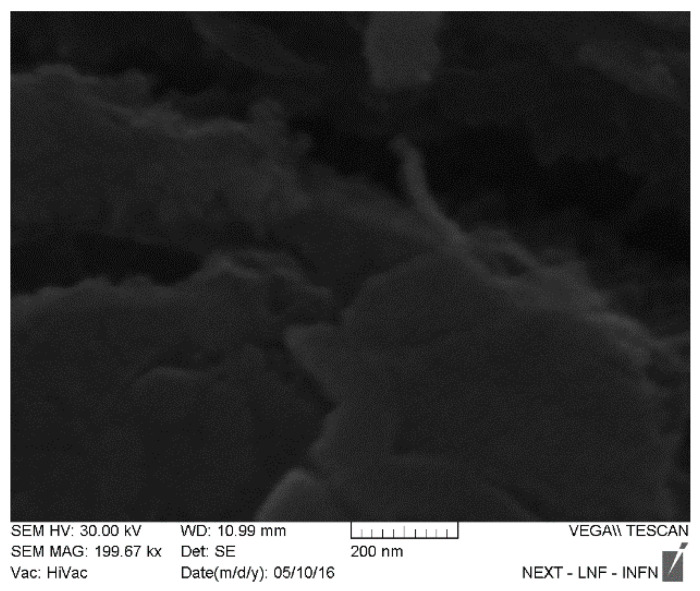
SEM image of CNT-NH_2_/Epoxy composite loaded at 2%w/w_._

**Figure A6:**
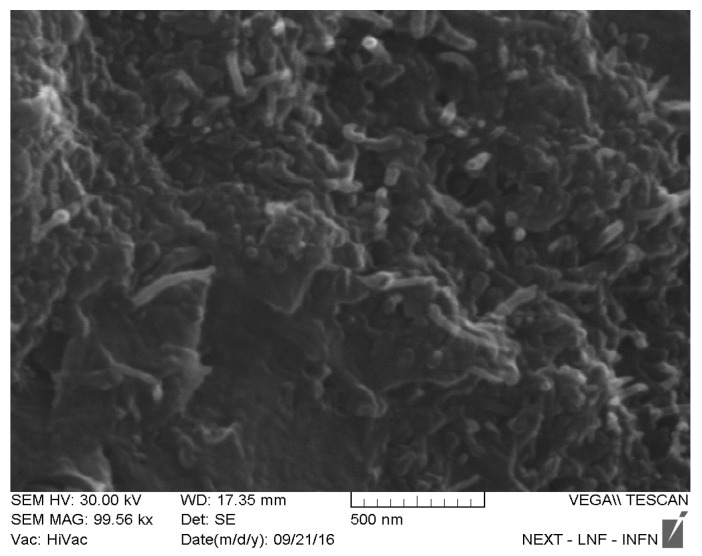
SEM image of CNT/Epoxy composite loaded at 10%_w/w._

**Figure A7:**
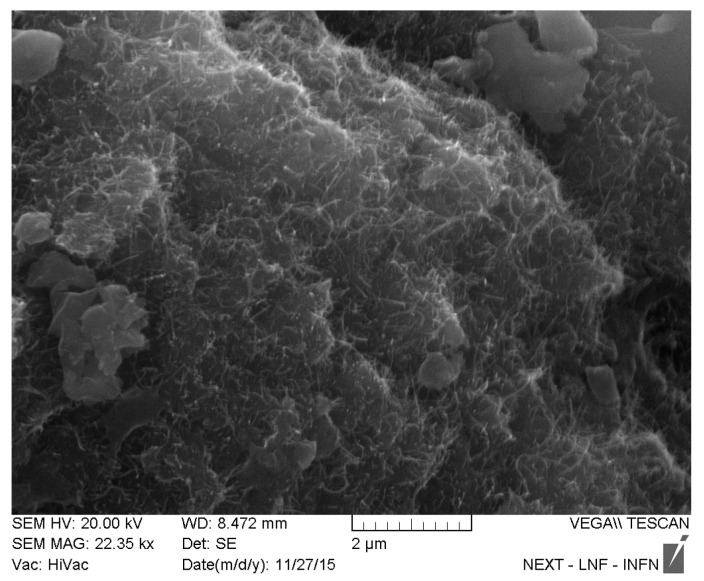
SEM image of CNT-NH_2_/Epoxy composite loaded at 10%_w/w._

**Figure A8:**
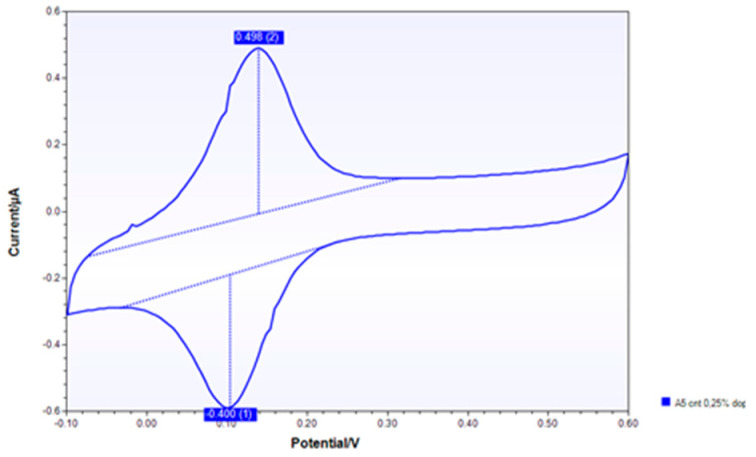
CV of CNT/epoxy composite, 0.25%, after 3 days in water.

**Figure A9:**
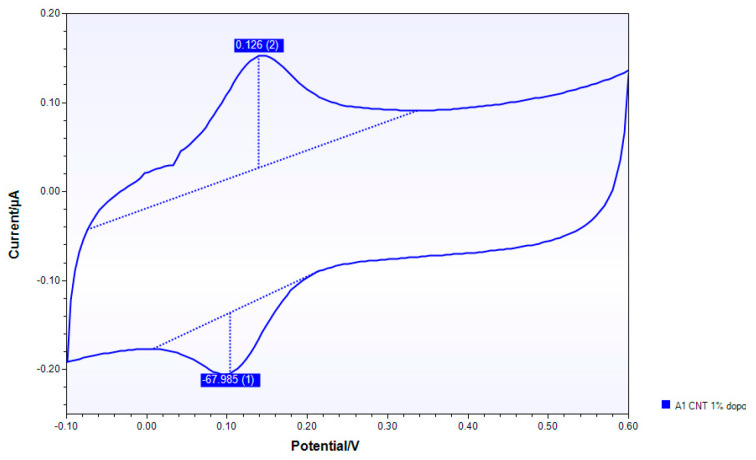
CV of CNT/epoxy composite, 1%, after 3 days in water.

**Figure A10:**
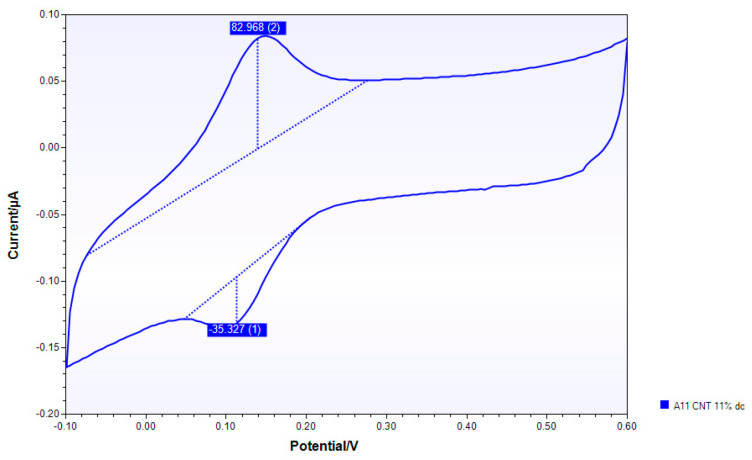
CV of CNT/epoxy composite, 2%, after 3 h in water.

**Figure A11:**
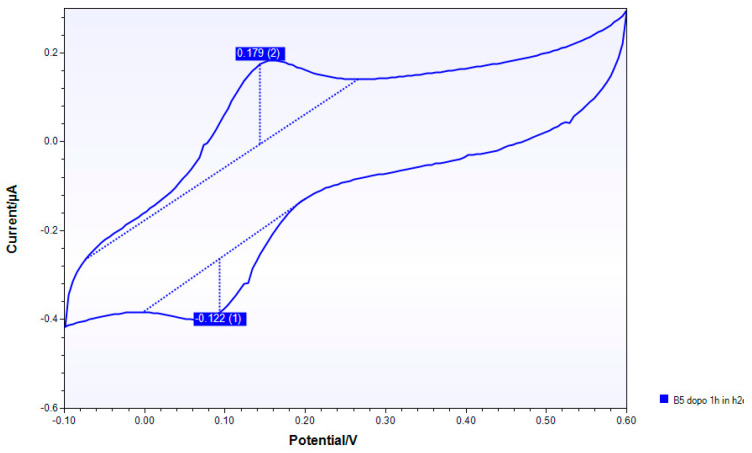
CV of CNT-NH_2_/epoxy composite, 0.25%, after 3 days in water.

**Figure A12:**
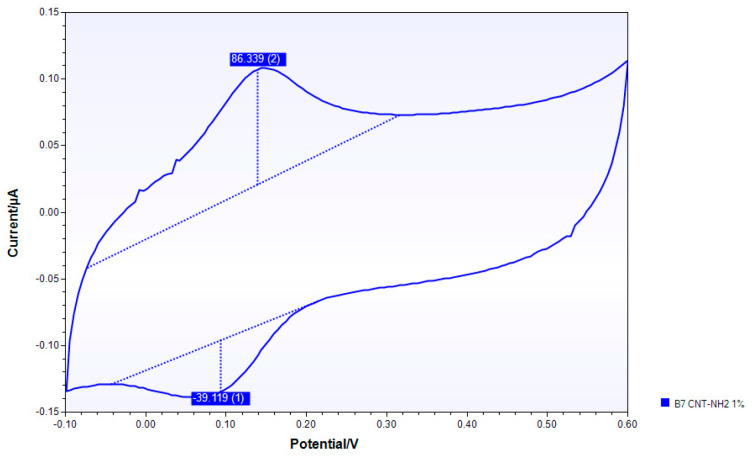
CV of CNT-NH_2_/epoxy composite, 1%, after 3 days in water.

**Figure A14:**
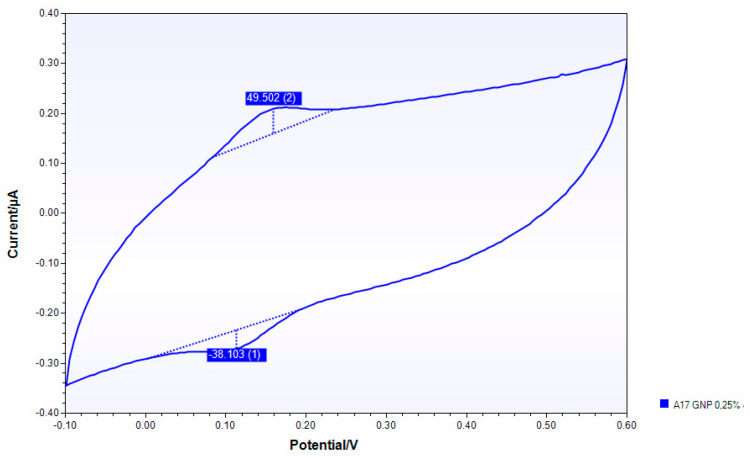
CV of GNP/epoxy composite, 0.25%, after 3 days in water.

**Figure A15:**
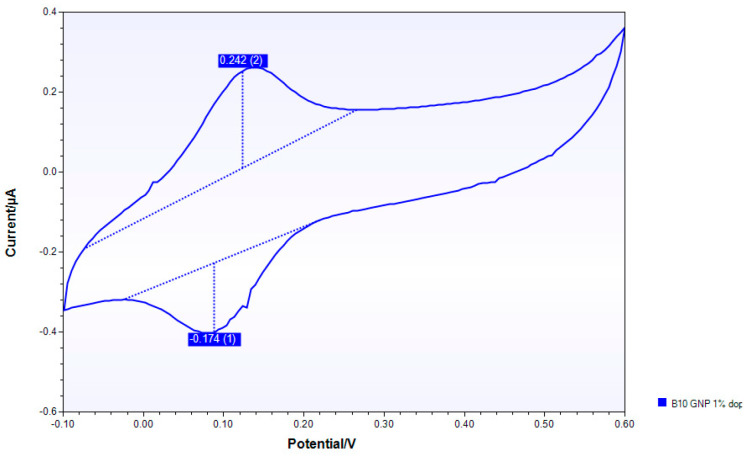
CV of GNP/epoxy composite, 1%, after 3 days in water.

**Figure A16:**
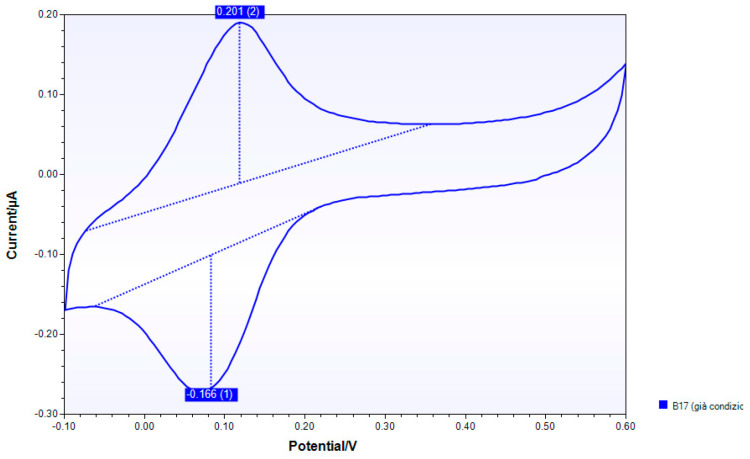
CV of GNP/epoxy composite, 2%, after 3 h in water.

**Figure A17:**
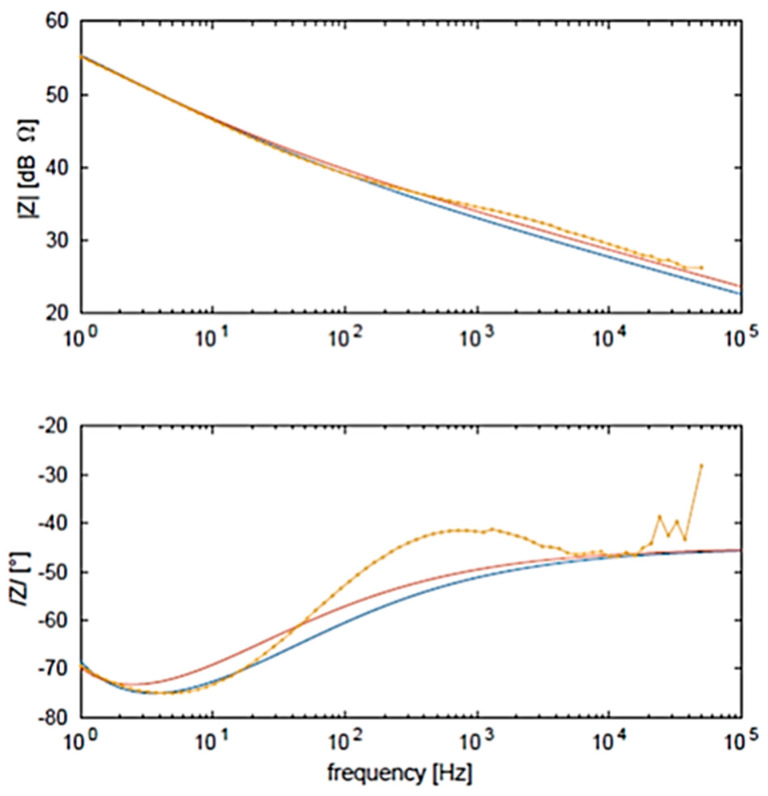
Best fit of neat epoxy resin for impedance (|Z|) and phase (°).

**Figure A18:**
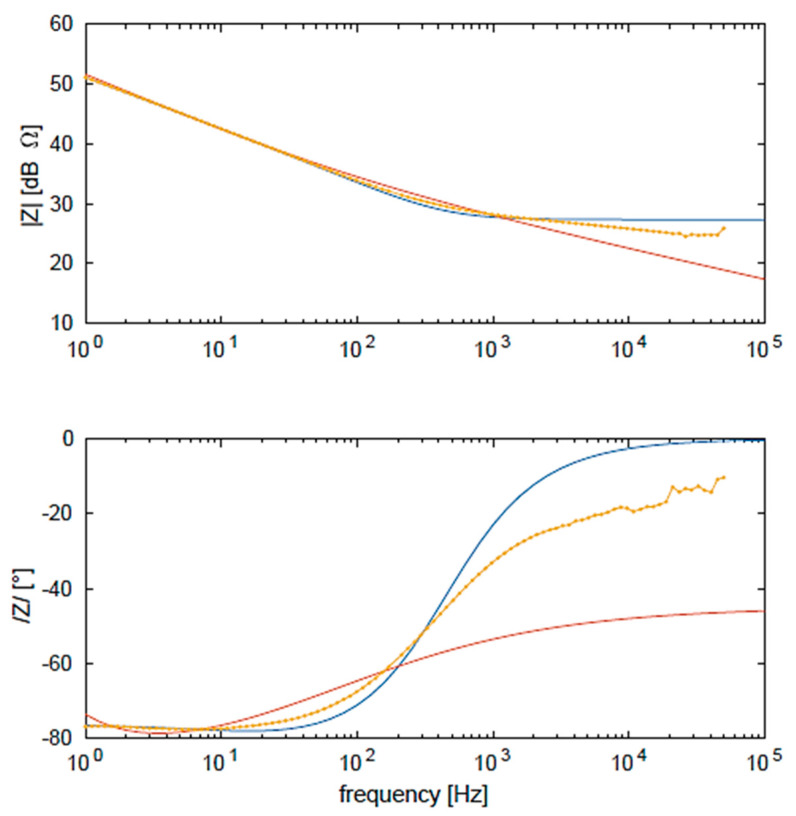
Best fit of neat epoxy resin for impedance (|Z|) and phase (°) after conditioning.

**Figure A19:**
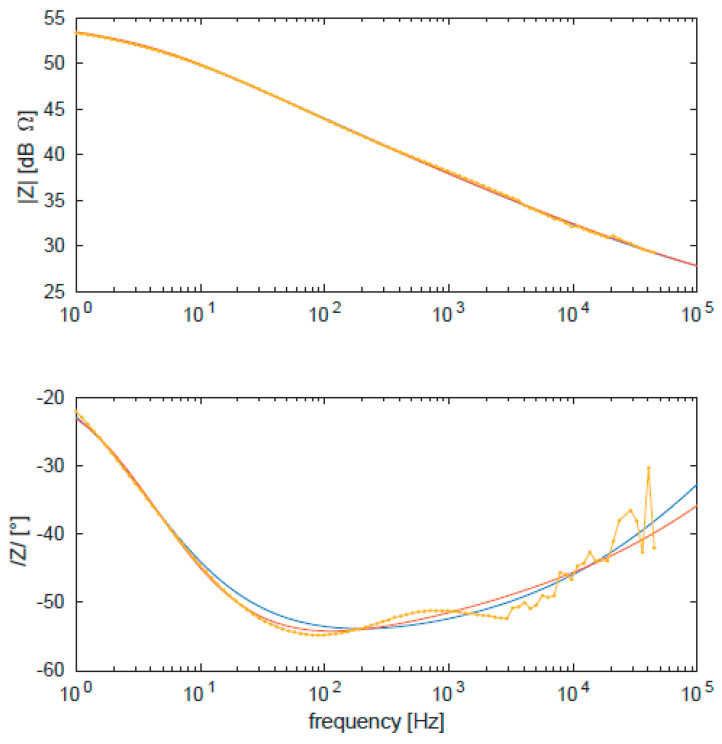
Best fit of CNT/epoxy 10% for impedance (|Z|) and phase (°).

**Figure A20:**
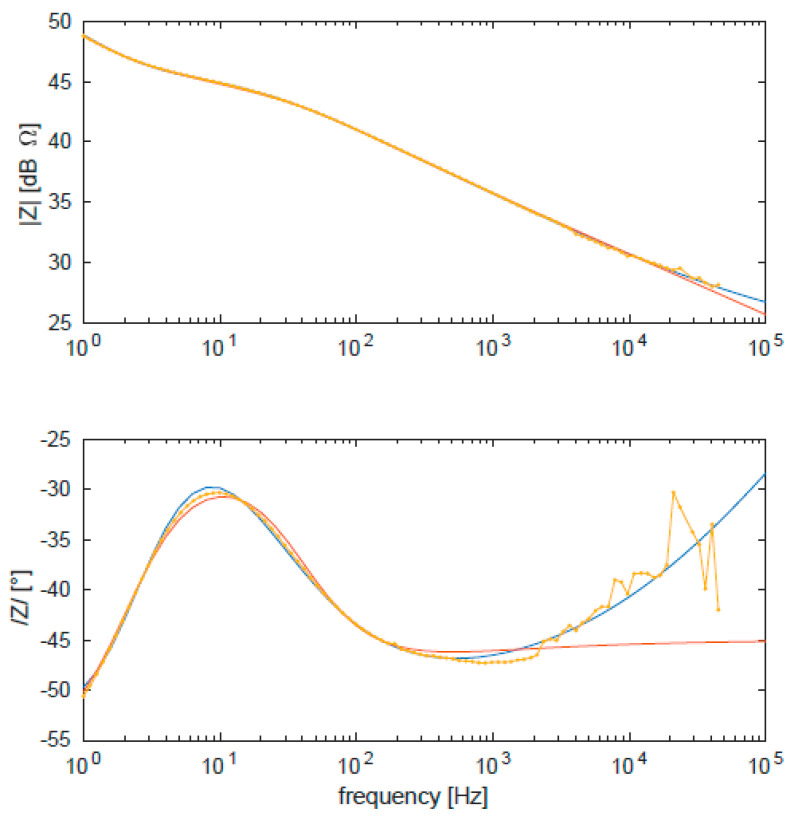
Best fit of CNT/epoxy 10% for impedance (|Z|) and phase (°) after conditioning.

**Figure A21:**
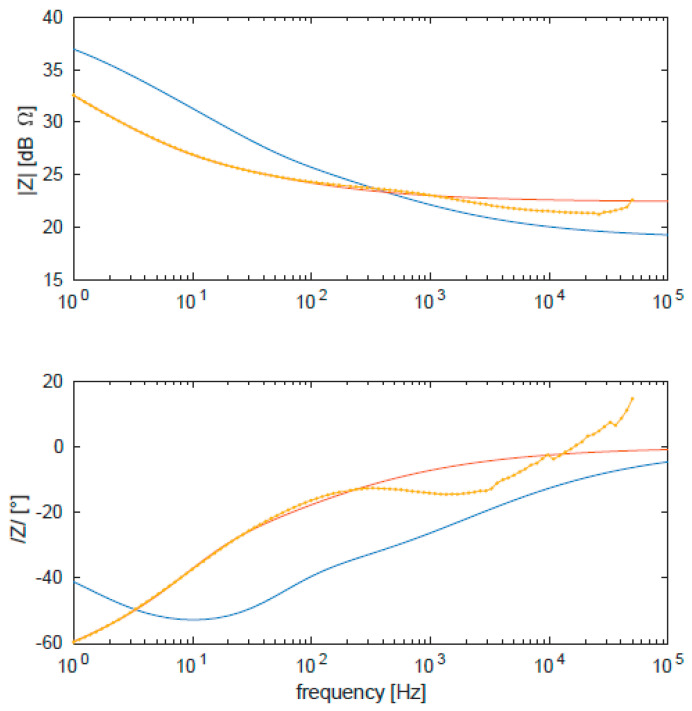
Best fit of GNP/epoxy 10% for impedance (|Z|) and phase (°).

**Figure A22:**
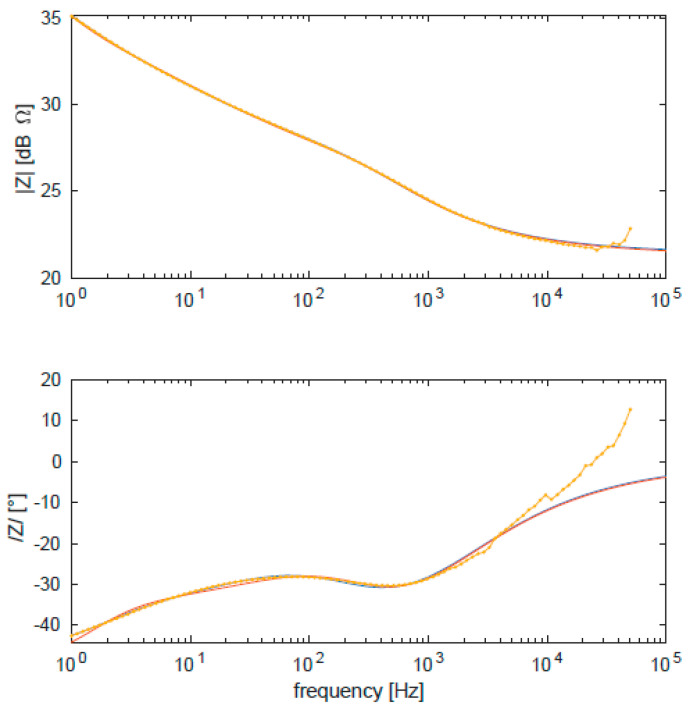
Best fit of CNT/epoxy 10% for impedance (|Z|) and phase (°) after conditioning.

**Figure A23:**
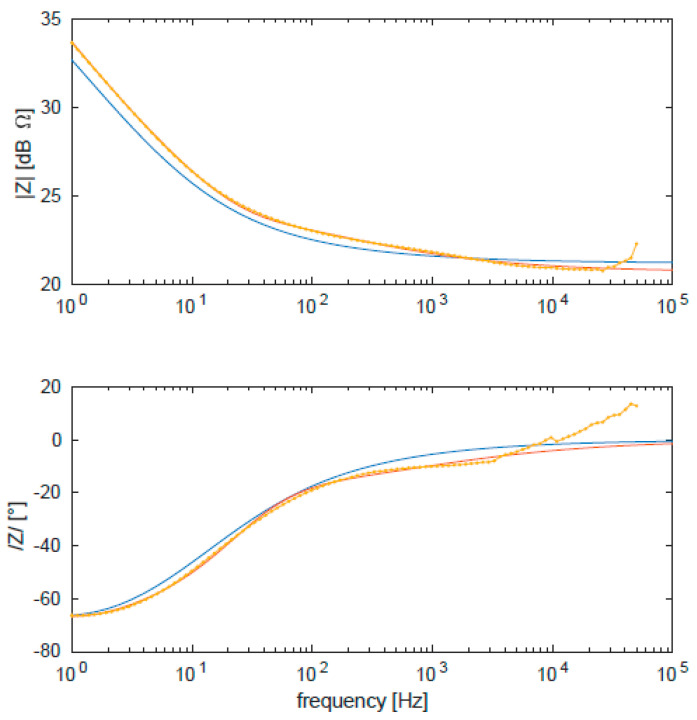
Best fit of CNT-NH_2_/epoxy 10% for impedance (|Z|) and phase (°).

**Figure A24:**
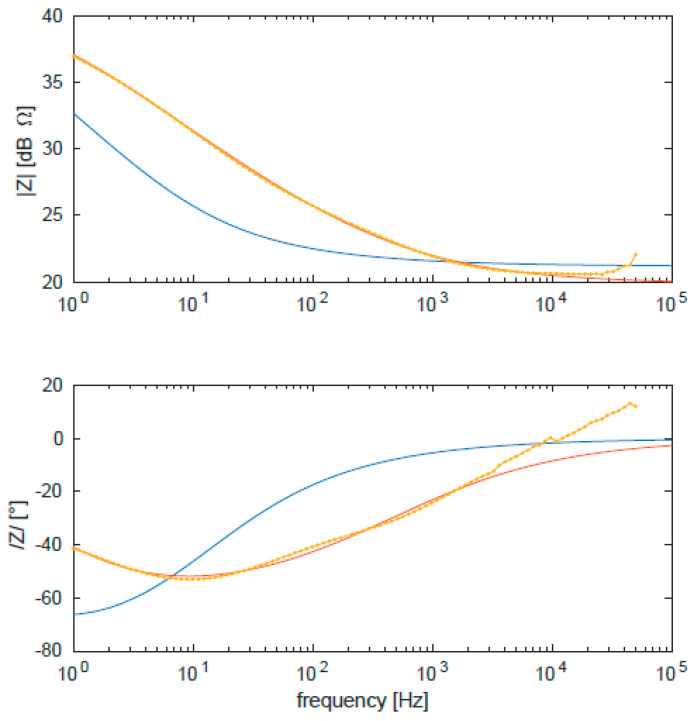
Best fit of CNT-NH_2_/epoxy 10% for impedance (|Z|) and phase (°) after conditioning.

**Figure A25:**
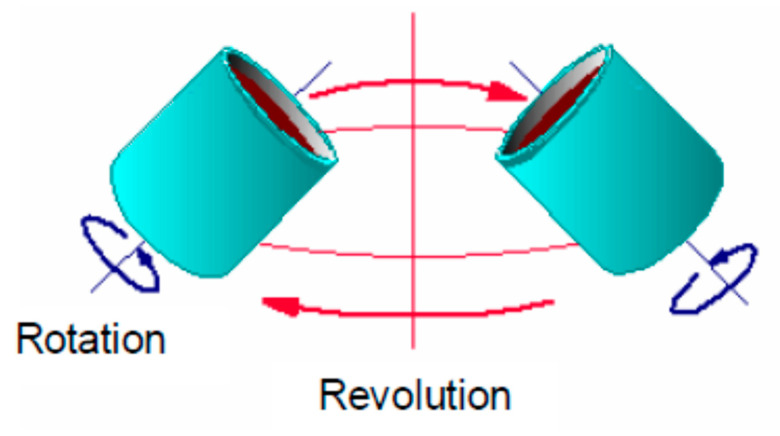
Representation of planetary motion.

## Appendix A

**SEM characterization**

## Appendix C

In the following figures we report the measured impedance (amber dots, outliers have been removed), together with the response of the electrical circuit as fitted by EIS Analyzer free software [17] (blue line) and by Matlab (red line).

## Appendix D

**Principle of ARV-310 working**

The Mixer “ARV-310”, provided by Thinky, is a planetary centrifugal mixer for mixing several phases (liquid or powder materials) and de-aerating them simultaneously.

The sample holder rotates while revolving in a set radius. This uninterrupted planetary motion generates a huge centrifugal force and relative shear stress, which compresses introduced air bubbles, out of the materials and mixes different phases at the same time. The presence of rotary pump helps the evacuation of the air from the sample. This pump generates a low vacuum down to few millibar.

In addition to mixing and de-aerating by rotation (max. 1000 rpm)/revolution (max. 2000 rpm), vacuum decompression achieves higher performance of de-aeration. Because of non-contact method (with no mixing blade), material deterioration can be prevented. High viscosity resins can be de-aerated in a short period of time without overflow. Highly constant reproducibility can be expected in mixing and de-aerating with no variation, neglecting in this way the operator-dependent errors.
